# Rhabdomyolysis in Community Acquired Bacterial Sepsis – A Retrospective Cohort Study

**DOI:** 10.1371/journal.pone.0007182

**Published:** 2009-09-29

**Authors:** Anita A. Kumar, Emmanuel Bhaskar, Ghanshyam Palamaner Subash Shantha, Porchelvan Swaminathan, Georgi Abraham

**Affiliations:** 1 Department of General Medicine, Sri Ramachandra University, Tamilnadu, Chennai, India; 2 Department of Biostatistics, BrainWave Bio Solutions Ltd, Tamilnadu, Chennai, India; 3 Department of Nephrology, Madras Medical Mission Hospital, Tamilnadu, Chennai, India; Fundação Oswaldo Cruz, Brazil

## Abstract

**Background and Objectives:**

Rhabdomyolysis is often associated with sepsis and gram positive bacterial pathogens are reported to be the most frequent cause of sepsis induced rhabdomyolysis. We report the pattern of infecting bacterial pathogens and associated causal factors in a South-Indian cohort.

**Design, Setting, Participants & Measurements:**

Retrospective cohort study of adult patients with community acquired bacterial sepsis complicated by rhabdomyolysis from March 2003 - August 2008. Rhabdomyolysis was defined as serum creatine kinase >2000 IU/L. The study population was divided into group-I (sepsis with gram positive pathogens), group–II (sepsis with gram negative pathogens) and group-III (culture negative sepsis).

**Results:**

103 patients (group I -15, group II- 34 and group III- 54) formed the study cohort. Mean age was 55 years and two-third had diabetes. Mean creatine kinase was 7114 IU/L and mean serum creatinine on admission was 2.4 mg/dl. Causative pathogen of sepsis was identified in 47.5%. Gram negative pathogens were more frequently (33%) associated with rhabdomyolysis than gram positive pathogens (14.5%). Lung was the commonest foci of sepsis (38.8%). 78.6% of the study population had one or more additional causal factor for rhabdomyolysis like statin intake, chronic alcoholism, hypokalemia, hypernatremia and hypophosphatemia. Mortality was 59%.

**Conclusions:**

Gram negative bacterial pathogens were more frequently associated with rhabdomyolysis than gram positive pathogens. Rhabdomyolysis in patients with sepsis is multifactorial and is associated with high mortality.

## Introduction

Rhabdomyolysis can be defined as disruption of skeletal muscle leading to leakage of intracellular muscle constituents especially myoglobin into the extracellular fluid [1]. Myoglobin is a heme containing compound with a molecular weight of 17,400 Daltons and may cause acute renal failure by tubular obstruction, toxicity of heme pigments, renal ischemia and decreased glomerular permeability [2]. Bywaters and Beal reported the first clinical syndrome compatible with rhabdomyolysis due to crush injuries in the year 1941 [3]. Though initially attributed to severe trauma etiology of rhabdomyolysis now includes severe physical exertion, drugs and toxins, muscle hypoxia, hereditary muscle enzyme defects, metabolic and endocrine disorders, infections and temperature alterations [4]. Acute renal failure complicates 10 to 67% cases of rhabdomyolysis and is an important cause of mortality [5]–[7].

The primary clinical event in the pathogenesis of rhabdomyolysis is elevated creatine kinase (CK) which is often used by clinicians to screen patients who are at risk for rhabdomyolysis. However the extent of CK elevation which may be associated with renal failure is not clear. Published reports have used a value of 1000 to 2500 IU/L (5 to 10 times the upper limit of normal) as a clinically significant elevation which may predispose to acute renal failure [8], [9]. But values of CK as low as 519 IU/L have been associated with marked elevation in serum myoglobin and biopsy proven renal failure due to rhabdomyolysis [10].

Sepsis is an important cause of morbidity and mortality [11]. Association between sepsis and rhabdomyolysis has been shown in multiple case reports [12]–[16]. But only a few original researches are available on the spectrum of infective pathogens associated with rhabdomyolysis [8]. This study was initiated to identify the epidemiology and laboratory profile of rhabdomyolysis due to bacterial sepsis in a developing nation and analyze the differences with published literature.

## Materials and Methods

### Study setting and design

Retrospective cohort study of adult patients with community acquired bacterial sepsis complicated by rhabdomyolysis from 2 tertiary care centers in South India from March 2003 to August 2008. The study was approved by the Sri Ramachandra University institutional review board. Written informed consent was obtained from all the study patients. For patients who did not survive when this retrospective study was conducted, a written and informed consent was obtained from the patients nearest relative. Sepsis was defined as systemic inflammatory response syndrome with history or clinical examination suggestive of infection [11]. Rhabdomyolysis was defined as creatine kinase (CK) more than 2000 IU/L (10 times the upper limit of normal). The presence of myoglobinuria was considered desirable but not mandatory for the diagnosis of rhabdomyolysis in our study cohort since myoglobin released after muscle injury circulates for a brief period (half life – 2 to 3 hours) and is rapidly excreted by the kidney [1] making estimation of serum or urine myoglobin a less sensitive test for the diagnosis of rhabdomyolysis. Patients with sepsis due to non-bacterial causes, hospital acquired sepsis, trauma, acute coronary syndrome, cerebrovascular accident, post-operative status, post-cardiac arrest, hyperglycemic crisis, hypothyroidism and malignant hyperthermia were not included.

### Method of patient evaluation

Hospital records of consecutive patients who satisfied the entry criteria were scrutinized for baseline characteristics (age, sex, current smoking status, current alcohol intake, diabetes mellitus, hypertension and current statin use), laboratory findings relevant to rhabdomyolysis (CK, urine myoglobin, admission electrolyte panel including calcium and phosphorus, serial serum creatinine values, blood gases, plasma lactate and uric acid), investigations to identify foci of sepsis (chest X-ray, ultrasound abdomen and cultures of blood, urine, cerebrospinal fluid, etc) and final outcome (death or survival). The study cohort was divided into 3 groups. Group-I (sepsis with gram positive pathogens), group–II (sepsis with gram negative pathogens) and group-III (culture negative sepsis).

### Statistical analysis

Baseline characteristics of study patients were expressed in number (%) for discrete variables, and as mean±standard deviation for continuous variables. Laboratory findings (CK, urine myoglobin, serum sodium, potassium, bicarbonate, calcium and phosphorus, serial serum creatinine values, blood gases, plasma lactate and uric acid) in different groups were compared using one-way analysis of variance or Kruskal Wallis analysis of variance. Multiple comparison between groups were done using Turkey HSD (honesty significance difference) test. A p value of <0.05 was considered statistically significant. All statistical analysis were done using SPSS version 15.

## Results

103 patients formed the study cohort. Baseline characteristics of the study patients are shown in **Table 1**. Mean age of our study cohort was 55 years with a predominant male distribution (60%). 47% were smokers and 41% were alcoholics. Nearly two-thirds (64%) were suffering from diabetes mellitus and more than a third (43%) had hypertension. 30% of our study cohort were on statins. Among diabetic patients 35% were on statins.

**Table 1:** Baseline characteristics of study cohort (n = 103).

Variable	Group-I	Group-II	Group –III	P value
	(n = 15)	(n = 34)	(n = 54)	
Age in years – mean±S.D	58±6	55±8	55±9	0.5
Sex				
Male- no (%)	11(73)	18 (53)	33 (61)	0.39
Female- no (%)	4 (27)	16 (47)	21 (39)	
Smoking- no (%)	11 (73)	11 (32)	26 (48)	0.02
Alcoholism- no (%)	5 (33)	14 (41)	23 (43)	0.8
Diabetes- no (%)	10 (67)	22 (65)	34 (63)	0.9
Hypertension–no (%)	6 (40)	16 (47)	22 (41)	0.82
Statin use -no (%)	4 (27)	11 (32)	16 (30)	0.91

Group –I: Gram positive sepsis.

Group-II: Gram negative sepsis.

Group-III: Culture negative sepsis.

The laboratory profile of the study cohort is shown in **Table 2**. The mean CK was 7114 IU/L and urine myoglobin was positive in 45%. Mean values of serum electrolytes were: sodium (Na) −136 meq/L, potassium (K) −3.7 meq/L, bicarbonate −13.3 meq/L (was <15 meq/l in 50%), calcium (Ca) −8.4 meq/L and phosphorus −4.5 meq/L. Mean serum creatinine on admission was 2.4 mg/dl and progressive increase in serum creatinine was observed in all groups. On admission the mean arterial Ph was 7.18 (46% had ph <7.2), mean lactate −2.8 meq/L and mean corrected anion gap −16 meq/L. 27.2% had hypokalemia defined as serum potassium <3 meq/l, 8% had hypernatremia defined as serum sodium >150 meq/l, 2.9% had hypophosphatemia defined as serum phosphate <2 mg/dl. None had severe hyponatremia defined as serum sodium <120 meq/l. 81 of 103 (78.6%) of our patients had one or more additional causal factor for rhabdomyolysis. The frequency of additional risk factor for rhabdomyolysis in our study patients is shown in **Table 3**. All patients received intensive care.

**Table 2:** Laboratory profile of the study cohort.

Variable	Group I	Group II	Group III	p value
Mean±S.D or no (%)	(n = 15)	(n = 34)	(n = 54)	
CK (IU/L)	8219±6022	8936±13109	5660±4131	0.18
Myoglobinuria	7 (47)	14 (41)	25 (46)	0.88
S.Na (meq/L)	132±8	136±7	137±9	0.15
S.K (meq/	3.9±1.7	3.6±1	3.7±0.9	0.6
S.Bicarbonate (meq/L)	14±3.6	13±4.7	13±4.7	0.4
S.Ca (mg/dl)	8±0.7	8.4±0.7	8.3±0.8	0.7
S.Phosporus (mg/dl)	4.7±1.1	4.2±1.1	4.5±1.1	0.34
S.Uric acid (mg/dl)	7.8±1.1	7.1±1.6	7.2±1.7	0.35
Anion gap (meq/L)	16±2.7	15±4	16±3.7	0.43
S. creatinine (mg/dl)				
Admission	2.5±1.1	2.3±0.6	2.5±1	0.71
Dialysis day	4.2±1.8	5.3±3	5.1±2.8	0.47
Arterial Ph	7.14±0.15	7.2±0.15	7.17±0.14	0.52
Plasma lactate(meq/l)	2.8±0.6	2.8±0.9	2.7±0.8	0.86

**Table 3:** Frequency of additional risk factors for rhabdomyolysis.

Risk factor	Frequency
Chronic alcoholism	41%
Statin intake	30%
Hypokalemia	27.2%
Hypernatremia	8%
Hypophosphatemia	2.9%

Analysis of variance revealed that there was no difference between the study groups (group-I to group-III) with respect to mean CK, frequency of myoglobinuria, admission creatinine, dialysis day creatinine, arterial Ph, plasma lactate, serum sodium, potassium, bicarbonate, calcium, phosphorus, uric acid and corrected anion gap (level of significance shown in **Table 2**). Further, multiple comparisons between groups by Turkey HSD test on laboratory values mentioned above showed no statistically significant differences.

Isolated pathogens and foci of sepsis among culture positive patients are shown in **Table 4**. Lung was the commonest foci (38.8%). The cause of sepsis could be identified in 47.5% of cases. Gram negative sepsis was more frequent (33%) than gram positive sepsis (14.5%). All patients required dialysis. 61 of 103 (59%) patients died.

**Table 4:** Isolated pathogens and foci of sepsis among culture positive patients (n = 49).

Foci of sepsis	Group I	Group II
	(n = 15)	(n = 34)
Lung (n = 20)	S. pneumoniae: 4	P. aerogenosa: 4
	S. aureus: 2	Enterobacter species: 1
	S. viridans: 1	K. pneumoniae: 5
		E. coli: 3
Meninges (n = 10)	S. aureus:1	E. coli: 2
	S. pneumoniae: 2	P. aerogenosa: 3
		P. mirabilis: 2
Urinary tract (n = 9)	Nil	P. aerogenosa: 3
		K. pneumoniae: 2
		E. coli: 3
		A. baumanni: 1
Endocardium (n = 6)	S. aureus: 3	E. coli: 2
	S. viridans: 1	
No foci (n = 4)	S. pneumoniae: 1	P. aerogenosa: 1
		E. coli: 2

S. pneumoniae: Streptococcus pneumoniae.

S. aureus: Staphylococcus aureus.

S. viridans: Streptococcus viridans.

P. aerogenosa: Pseudomonas aerogenosa.

K. pneumoniae: Klebsiella pneumoniae.

E. coli: Escherichia coli.

P. mirabilis: Proteus mirabilis.

A. baumanni: Acinatobacter baumanni.

## Discussion

Infections accounted for 5% of rhabdomyolysis in the literature 25 years ago [17], [18]. Betrosian et al. [8], using a strict entry criteria for rhabdomyolysis, compared the pattern of rhabdomyolysis due to bacterial sepsis (n = 35) with patients having sepsis without rhabdomyolysis (n = 122) and observed that infections with gram positive pathogens (especially Staphylococcus aureus and Streptococcus faecalis) more frequently caused rhabdomyolysis than gram negative pathogens with lung being the foci of sepsis in most patients (34%) followed by urosepsis, gall bladder infection, pancreatitis and catheter related infections. In contrast our study has shown the dominance of gram negative pathogens (especially Pseudomonas, E.coli and Klebsiella) over gram positive pathogens. Similar to their study lung was the most frequent (38.8%) foci of sepsis in our cohort. We did not observe gall bladder infection or pancreatitis probably due to selection bias as these patients are predominantly managed by surgeons in our study centers.

Further, their study [8] used a CK level more than 2500 IU/L as definition for rhabdomyolysis and excluded patients with acidosis defined as serum bicarbonate less than 15 meq/L in addition to alternative causes for rhabdomyolysis (trauma, surgery, dysnatremia, hypokalemia, hypophospatemia) since they were attempting to correlate plasma osmolality with levels of CK. We did not exclude patients with bicarbonate less than 15 meq/L, dysnatremia, hypokalemia and hypophosphatemia since these abnormalities commonly co-exist with sepsis and our objective was to identify all contributing factors to rhabdomyolysis in patients with sepsis. Furthermore the baseline characteristics of their study cohort [8] with reference to co-morbidities like diabetes, hypertension, chronic alcohol intake, concomitant statin intake is not known. It should be noted that significant number of our patients were suffering from diabetes (64%) and hypertension (43%). Given this fact it is not surprising that 30% of our study patients were on statins which can independently cause rhabdomyolysis. Further our patients had co-existing causal factors for rhabdomyolysis like chronic alcoholism (41%), hypokalemia (27.2%), hypernatremia (8%) and hypophosphatemia (2.9%). The predominance of gram negative pathogens in our study can be partially explained by the high proportion of diabetic patients.

The proposed mechanism of rhabdomyolysis due to sepsis are (i) direct muscle invasion by the pathogen, (ii) toxin generation, (iii) cytokine mediated muscle cell toxicity, and (iv) muscle ischemia due to shock [4], [17], [19]–[23]. Rhabdomyolysis due to direct muscle invasion and toxin generation is classical with Staphylococcus aureus [24]. Streptococci, Salmonella and Staph. aureus have been demonstrated in muscle biopsies of patients with rhabdomyolysis while Legionella, a frequent pathogen associated with rhabdomyolysis has not been demonstrated in muscle biopsies indicating a probable distant mechanism [4].

The mean CK and presenting biochemical investigations between our sub-groups showed no significant differences. This emphasizes the fact that despite different possible mechanisms adopted by microbes the pattern of final clinical presentation of rhabdomyolysis is similar.

The pathogens identified in our study are identical to previous published literature except Acinetobacter which has not been previously reported. Bacterial pathogens associated with rhabdomyolysis are Legionella, Streptococcus pneumoniae, Staphylococcus aureus, Streptococcus viridans, Salmonella species, Staphylococcus epidermidis, Francisella tularensis, Streptococcus faecalis, Meningococci, Hemophilus influenza, E.coli, Pseudomonas, Klebsiella, Enterococcus faecalis and Bacteroides [4], [8], [10], [20], [25], [26]. Lung was the most frequent (38.8%) foci of sepsis in our study. This is consistent with previous reports which show a close relation between lung sepsis and rhabdomyolysis [8], [27], [28].

As discussed earlier statins, chronic alcoholism, hypokalemia, hypernatremia and hypophosphatemia were additional causes which could have facilitated the occurrence of rhabdomyolysis in our study cohort. Combination of causal factors in patients with rhabdomyolysis is well known [17].

Statins are supposed to induce defective glycoprotein synthesis in muscle membrane, deficient chloride channel activation in muscle membrane and increased intracellular calcium leading to impaired membrane function, all leading to myocyte injury [29]. Our study cohort is notable for the high proportion of diabetics and 35% of them were on statins. The possibility of statin intake precipitating rhabdomyolysis in diabetic patients with sepsis cannot be overlooked since diabetes is a known risk factor for statin induced myopathy [30].

Rhabdomyolysis due to acute alcoholism is well known [4]. Rhabdomyolysis may also occur secondary to chronic alcohol intake [31]. The proposed mechanism for rhabdomyolysis due to chronic alcoholism are electrolyte disturbances like hypokalemia, hypophosphatemia and hypomagnesemia as evidenced by marked depletion of intracellular potassium, phosphorus and magnesium in skeletal muscles of chronic alcoholics and experimental animals fed with alcohol [32], [33]. Potassium depletion can decrease glycogen synthesis within muscle and also impair potassium dependent vasodilatory response [34]. This combination of diminished glycogen reserve and impaired oxygen delivery may lead to ATP depletion producing muscle injury [4]. Hypophosphatemia may also limit ATP production and produce an energy deficient state leading to muscle injury [4].

The mortality in our study cohort was 59%. Since our study cohort had frequent co-morbid conditions the mortality observed cannot be interpolated to a relatively healthy population. All our study patients received hemodialysis probably due to the presence of multiple disorders and progressive raise in serum creatinine warranting aggressive management by the treating physician to avoid unfavorable outcomes. We preferred not to analyze the factors contributing to death since treatment of rhabdomyolysis varied between physicians in our study. Though alkalinization of urine and diuretics are frequently used to treat rhabdomyolysis their efficacy in preventing progression of renal failure is unknown. Recent evidence highlights the lack of efficacy and possible harm with bicarbonate and diuretics [35].

Our study did not analyze the severity of illness using Acute Physiology and Chronic Health Evaluation (APACHE) score or a comparable score. This is an important limitation of the study since this information would have shed more light on disease severity on admission and during hospital stay. Since the study primarily looked at the etiology of bacterial sepsis and not the risk factors for poor outcome, lack of these details may not affect the primary objective of the study.

In conclusion, our study observed that association between rhabdomyolysis and sepsis is relatively common and is associated with additional causal factors for rhabdomyolysis like statin use, chronic alcohol intake, hypokalemia, hypernatremia and hypophosphatemia. Lung was the most frequent septic foci and we observed that gram negative organisms were more frequent than gram-positive pathogens. Furthermore, our data questions the safety of continuing statins in diabetic and alcoholic patients with active sepsis.
